# Factors Associated with Survey Non-Response in a Cross-Sectional Survey of Persons with an Axial Spondyloarthritis or Osteoarthritis Claims Diagnosis

**DOI:** 10.3390/ijerph17249186

**Published:** 2020-12-09

**Authors:** Johanna Callhoff, Hannes Jacobs, Katinka Albrecht, Joachim Saam, Angela Zink, Falk Hoffmann

**Affiliations:** 1 Epidemiology, German Rheumatism Research Centre, 10117 Berlin, Germany; albrecht@drfz.de (K.A.); zink@drfz.de (A.Z.); 2 Department of Health Services Research, Carl von Ossietzky University Oldenburg, 26129 Oldenburg, Germany; hannes.jacobs@uni-oldenburg.de (H.J.); falk.hoffmann@uni-oldenburg.de (F.H.); 3 Department Medicine and Health Services Research, BARMER Institute for Health System Research, BARMER, 73525 Schwäbisch-Gmünd, Germany; Joachim.Saam@barmer.de; 4 Department of Rheumatology and Clinical Immunology, Charité University Medicine Berlin, 10117 Berlin, Germany

**Keywords:** survey non-response, claims data, health services research, osteoarthritis, spondyloarthritis, non-response bias

## Abstract

Non-response in surveys can lead to bias, which is often difficult to investigate. The aim of this analysis was to compare factors available from claims data associated with survey non-response and to compare them among two samples. A stratified sample of 4471 persons with a diagnosis of axial spondyloarthritis (axSpA) and a sample of 8995 persons with an osteoarthritis (OA) diagnosis from a German statutory health insurance were randomly selected and sent a postal survey. The association of age, sex, medical prescriptions, specialist physician contact, influenza vaccination, hospitalization, and Elixhauser comorbidity index with the survey response was assessed. Multiple logistic regression models were used with response as the outcome. A total of 47% of the axSpA sample and 40% of the OA sample responded to the survey. In both samples, the response was highest in the 70–79-year-olds. Women in all age groups responded more often, except for the 70–79-year-olds. Rheumatologist/orthopedist contact, physical therapy prescription, and influenza vaccination were more frequent among responders. In the logistic regression models, rheumatologist/orthopedist treatment, influenza vaccination, and physical therapy were associated with a higher odds ratio for response in both samples. The prescription of biologic drugs was associated with higher response in axSpA. A high Elixhauser comorbidity index and opioid use were not relevantly associated with response. Being reimbursed for long-term care was associated with lower response—this was only significant in the OA sample. The number of quarters with a diagnosis in the survey year was associated with higher response. Similar factors were associated with non-response in the two samples. The results can help other investigators to plan sample sizes of their surveys in similar settings.

## 1. Introduction

In epidemiology and health services research, surveys are an important data collection method. Survey non-response is unavoidable and may lead to great challenges in the conduct, analysis, and interpretation of a survey [1,2]. It can threaten the validity of a study in the presence of non-response bias [3]. While there are reasons for non-response that are not connected to the research question of a health survey, e.g., that the participants just have no time to answer [4], there are many reasons that are connected to the content. Those can be hospitalizations in persons with a more severe disease or a higher motivation to participate in research in sicker persons. However, such information on potential non-response bias are often not available.

In contrast to that, in routinely collected healthcare data, there is no non-response bias, because all data are available for the whole population under risk [5]. Claims data of health insurance funds are increasingly used as a source in epidemiology and health services research in Germany [6,7,8]. They contain information about ICD-10 diagnosis, prescription of medication, physical therapy and medical aids, age and sex, hospitalization, and information on the contact to physicians and their medical specialty. However, claims data cannot offer information on disease activity, clinically validated diagnoses, or patient-reported outcomes [9]. The latter are critical in studies on health care provision in chronic conditions such as musculoskeletal diseases. Several workgroups attempted to overcome this limitation by predicting disease severity with the use of claims data, often using data on prescribed medication [10,11], but the prediction was only moderately good [10,12].

The combination of survey and claims data allows utilizing the strengths of both data sources: claims data contain information on diagnoses, age, and sex as well as services used. Linked survey data can add disease severity and patient-reported outcomes. Such a linked data source also allows for a comparatively thorough investigation of factors that are associated with non-response. While the association of sex and age with non-response has been investigated extensively, other factors were not studied this often. In the Diadec study [13], Linnenkamp et al. 2020 surveyed persons with a diabetes diagnosis in German claims data. They found an age–sex interaction in the analysis of factors associated with response and higher medication utilization among responders. However, it is not clear whether results are comparable to other chronic diseases. Therefore, we performed a similar analysis for persons with two different musculoskeletal diseases, axial spondyloarthritis (axSpA) and osteoarthritis (OA).

AxSpA is an inflammatory musculoskeletal disease that primarily affects the spine and joints [14]. OA is a degenerative joint disease that can lead to pain and stiffness of the joints [15]. Patients are mainly treated by rheumatologists in axSpA and by orthopedists in OA. Standard treatment options are non-steroidal anti-rheumatic drugs (NSAIDs), for all axSpA [14] and OA patients [16] and tumor necrosis factor alpha inhibitors (TNFi) for severe cases of axSpA. Opioids and other agents might also be prescribed when further pain relief is needed.

We could not identify any analyses of non-response to surveys in patients with axSpA. In OA, Roddy et al. 2015 [17] were able to compare the age and sex of responders and non-responders to a survey in persons aged 50 years and older with radiographic foot OA. Overall, response rates were a little bit higher for women (57%) than men (53%). In men, but not in women, there was a different response in the age groups. In the Knee Clinical Assessment Study [18], patients were recruited from three clinics. In this study, also, only age and sex were analyzed for non-responders and other variables, indicating that a potential non-response bias was not available in both studies. We aim to close this gap and describe factors associated with non-response using claims data linked with survey data in two different musculoskeletal diseases.

The aims of this analysis were to:

- Characterize respondents and non-respondents of the two surveys with respect to relevant characteristics available from the claims data (age, sex, health care utilization).
- Compare differences in the response patterns among the two diagnoses and discuss the results in comparison to the diabetes sample in order to assess the generalizability of the results to other chronic diseases.

## 2. Materials and Methods

### 2.1. Sample Selection and Survey Procedure

This was a cross-sectional study. We used data from the PROCLAIR project (Linking Patient-Reported Outcomes with Claims Data for Health Services Research in Rheumatology) which included access to data from a large German statutory health insurance (“BARMER”). This was a collaborative project funded by the German Ministry of Education and Research. Researchers from the German Rheumatism Research Centre, the Carl von Ossietzky University of Oldenburg, the Charité-Universitätsmedizin Berlin, and the Carl Gustav Carus University of Dresden and the BARMER statutory health insurance participated in the project. Germany has a universal health care system with the majority of the population being insured by a statutory health insurance. The BARMER is one of the largest health insurance companies in Germany and covers the whole country. For billing purposes, claims data are routinely collected by the health insurance. The clinicians who provided care for the patients were only indirectly involved in the project by providing usual care.

The aims of the PROCLAIR project were to gain knowledge about axSpA and OA patients from the general population, especially those without contact to specialized physicians [19,20]. To achieve this, we utilized a linked dataset of claims data and survey data. Two stratified random samples (axSpA, ICD-10 Code M45, OA of the hip, knee or polyarticular OA (ICD-10 codes M15-17)) were selected from the population of over 7 million insurants aged 18–79 who were continuously insured in 2013 and 2014. Persons with the respective claims diagnosis in at least two quarters of the sample year were eligible for selection. The axSpA sample was stratified by age and sex. The OA sample was stratified by age, sex, and diagnosis (polyarticular OA, hip OA, knee OA). For each axSpA stratum, 500 persons were selected, and for each OA stratum, 330 persons were selected, except for the stratum with polyarthritic men aged 30–39, which only contained 134 persons (full sample). Samples were randomly selected using the SURVEYSELECT procedure in SAS 9.4 software. In both samples, the persons who were still insured at the time of the survey were contacted by mail. The letter was sent by the health insurance directly to the insurants. It contained the following parts:

- An introduction to the project with explanation of the data protection measures and an invitation to participate in the research project.
- A form to declare consent to participate.
- The study questionnaire (6 pages for the axSpA sample, 7 pages for the OA sample).
- A stamped envelope to send the consent form to the health insurance.
- A stamped envelope to send the questionnaire to the German rheumatism research center.

It was explained that nobody outside the health insurance will know the names of the participants and that nobody from the health insurance will get access to the questionnaires. 

One reminder was sent for each survey. The reminder contained a modified invitation letter to participate. All other contents were similar to the original mailing. Specifically, no incentives were offered to the participants. For the responders, the date of the postmark was recorded. Data collection for the axSpA sample lasted from 16 November 2015 to 1 June 2016 and from 3 June 2016 to 31 December 2016.

Details concerning the axSpA sample are published in [20]; those concerning the OA sample are published in [19].

### 2.2. Claims Data Available for Analysis

Characteristics of responders and non-responders were compared. The following variables from the claims data were used: age, sex, information on therapy identified via anatomical therapeutic chemical (ATC)-codes: NSAIDs (M01A), biologic disease-modifying anti-rheumatic drugs (bDMARDs) including TNFi (L04AB; L04AA24,26; L01XC02; L04AC03, 07, 08, 10, 13–15), opioids (N02A), number of all prescribed distinct medications (as a proxy measure of comorbidity), seeing a specialist (rheumatologists/orthopedists), Elixhauser comorbidity score [21], influenza vaccination (as a proxy measure of health behavior), proportion of persons with hospitalization, persons with a level of care (which means being eligible to receive reimbursement for professional long-term care, the variable was coded as binary variable), prescription of physical therapy. The number of quarters with at least one diagnosis of axSpA or OA in the year of the survey was calculated as a coarse proxy for disease severity. All claims data were evaluated for the year when the survey was conducted (2015 for axSpA, 2016 for OA).

### 2.3. Statistical Analysis

The goals of the PROCLAIR project were to identify possible differences e.g., in work ability among subgroups of the sample. The aim was to be able to detect a relatively small effect size of 0.25 at an error-1-level of 5% and with a power of at least 85% in a two-tailed *t*-test. This results in a sample size of 289 per group. We assumed that 60% of the contacted persons respond. This resulted in a sample size of 482 per group, which was rounded to a sample size of 500 in the axSpA sample. In OA, samples were stratified for the type of OA. We assumed that there would be considerable overlap between knee, hip, and polyarthritic OA with 20% of the knee OA patients also having hip OA and 20% also having polyarthritic OA and vice versa. As a result of that, the sample sizes for the OA subgroups were n = 330 so that there would still be around 500 patients with the respective OA type in the sample. All analyses were weighted to represent the whole population of insurants with axSpA or OA in the claims data. The SAS survey procedures were used to obtain frequencies and means. Factors associated with response were assessed in one multiple logistic regression model for each sample. Variables to be included in the model were pre-selected using clinical expertise. Both the axSpA and the OA model included the following variables: age (in categories matching the age strata), sex, the interaction term of age and sex, the Elixhauser comorbidity index, influenza vaccination, level of care present (yes/no), physical therapy prescription, and the number of quarters with a diagnosis in the survey year. The axSpA model additionally included bDMARD prescription and rheumatologist contact, while the OA model additionally included opioid prescription and orthopedist treatment. Odds ratios (OR), 95% confidence intervals (CI), and corresponding p-values were obtained. The response times were calculated as the time between the first dispatch of the survey and the postmark on the return envelopes. There were few (n = 14) missing values for the response time in the OA sample, which were excluded from the response time analysis. As they were assessed from claims data, all other variables had no missing values. Ethical approval was obtained from the ethics committee of the Charité–Universitätsmedizin Berlin in March of 2015 (EA1/051/15). This research was conducted in agreement with the Declaration of Helsinki.

## 3. Results

### 3.1. Response by Age and Sex

**Figure 1:**
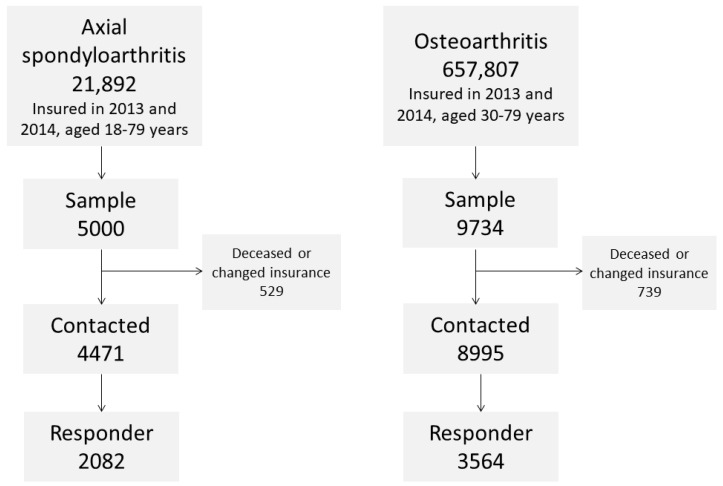
Flowchart showing sample sizes in the surveys.

A total of 21,892 individuals aged 18 to 79 years had an axSpA diagnosis in the claims data (Figure 1).

**Figure 2:**
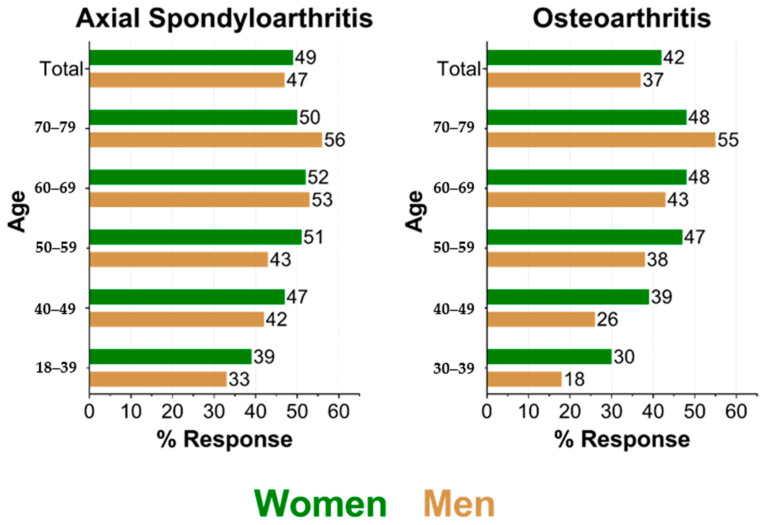
Response in percent by age stratum and sex for both samples.

Of those, 4471 were contacted and 2082 (47%) responded. A total of 657,807 individuals aged 30 to 79 years had an OA diagnosis. Of those, 8995 were contacted and 3564 (40%) responded. The response was generally higher among older persons in both diseases (Figure 2).

The response was higher for women except in older persons in both diseases. Response ranged from 18% in 30–39-year-old men with OA to 56% in 70–79-year-old men with axSpA.

### 3.2. Differences between Responders and Non-Responders

Differences between the responders and non-responders are shown in Table 1. Responders had higher frequencies of prescriptions than non-responders for NSAIDs (58% vs. 50% in axSpA, 48% vs. 40% in OA) as well as for bDMARDs in axSpA (14% vs. 9%). Opioid prescription frequencies did not differ relevantly. In responders, more persons with an axSpA claims diagnosis were seeing a rheumatologist (33% vs. 21%), and more persons with an OA diagnosis were seeing an orthopedist (57% vs. 44%). Comorbidities measured by the Elixhauser score did not differ between the groups. Responders had slightly more prescribed medications (median 6.0 vs. 5.0 in axSpA, 6.4 vs. 5.8 in OA). Relevant differences were found in the percentages of persons with an influenza vaccination (33% vaccinated vs. 22% in axSpA, 39% vs. 29% in OA). In OA, but not in axSpA, there were more persons with a level of care among the non-responders. In both diseases, the response was higher in persons for whom the respective diagnosis was documented in all four quarters of the survey year as well as in those who had prescriptions of physical therapy.

Table 2 shows the results of the regression models for axSpA and OA. In the multiple logistic regression model for axSpA, there were several variables associated with response. Persons in the age groups of 50–59, 60–69, and 70–79 years all had higher ORs of response than those in the 18–39 years comparator group. In addition, higher ORs for response were found in persons in rheumatologic treatment (1.71; 95% CI 1.46; 2.01), persons who received an influenza vaccination (1.32; 95% CI 1.13; 1.55), physical therapy (1.42; 95% CI 1.25; 1.62) or bDMARDs (1.28 (1.02; 1.59), and persons with more quarters with a diagnosis of axSpA in the survey year (1.18; 95% CI 1.12; 1.25). Male sex was associated with a lower OR for response (0.75; 95% CI 0.56; 1.00) as well as a higher Elixhauser index (0.95 per comorbidity, CI 0.92; 0.98). The level of care seemed to be associated with response, but the point estimate for the OR had a wide confidence interval. Supplementary Table S1 shows comparisons for the odds to response between women and men given the respective age groups. In the 18–39, 40–49, and 50–59 year age groups, women had a higher OR for response than men, while in the 70–79 years group, women had a lower OR for response (0.73; 95% CI 0.56; 0.97). The difference was statistically significant for age groups 18–39 and 70–79.

In the OA model, compared to the 30–39-year-olds, all other age groups had higher ORs of response. Being in orthopedic treatment (OR 1.56; 95% CI 1.37; 1.78), influenza vaccination (1.40; 95% CI 1.22; 1.61), and physical therapy prescription (1.18; 95% CI 1.03; 1.35) were all associated with higher response. Male sex was associated with lower response (0.51 95% CI 0.38; 0.67) as well as having a level of care (OR 0.43 95% CI 0.30; 0.62). Opioid prescription, Elixhauser comorbidity index, and the number of diagnoses in the survey year were not associated with response. Similarly to the axSpA sample, in the age groups of 30–39, 40–49, and 50–59 years, women had higher ORs for response (ORs between 1.36 and 1.97, all statistically significant, Supplementary Table S1), while among the 70–79-year-olds, women had a lower OR for response than men (0.76; 95% CI 0.63; 0.93), which was also statistically significant.

### 3.3. Analysis of the Response Time

**Figure 3:**
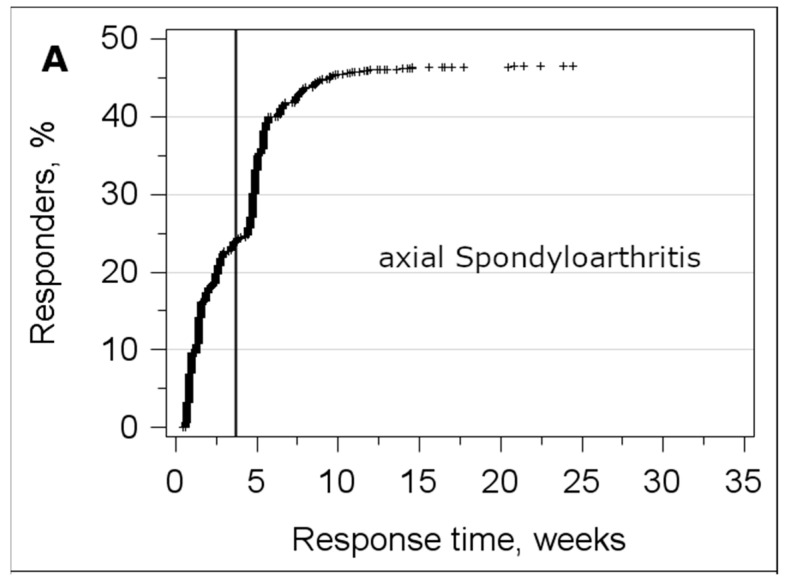

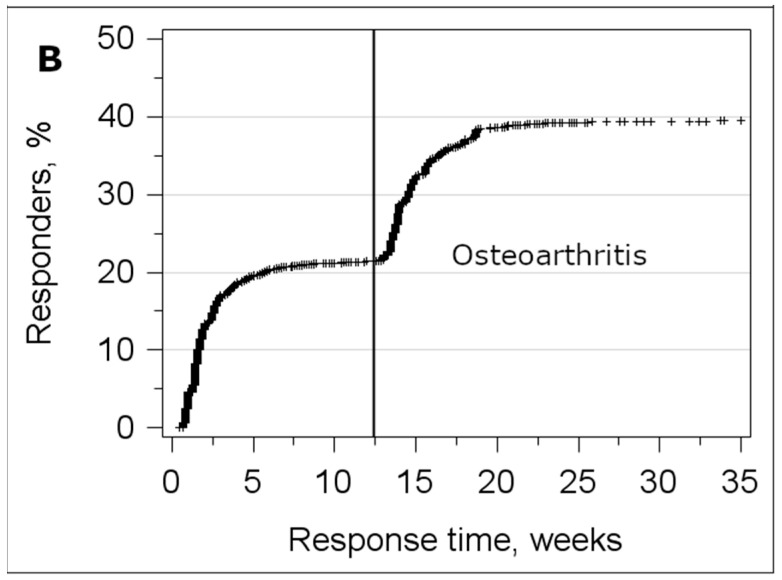
Title: Response by time. Legend: Proportion of responders by response time for (**A**) axial spondyloarthritis (n = 2082) and (**B**) osteoarthritis (n = 3550). The vertical lines mark the time when the reminders were sent.

In the axSpA sample, a reminder was sent 4 weeks after the first dispatch of the survey. Due to the holidays, the reminder in the OA sample was sent after 12 weeks. Figure 3 shows the percentage of responders by response time. In both samples, about half of the responders returned the survey before and the other half after the reminder was sent.

## 4. Discussion

Factors associated with survey non-response were analyzed in two claims data samples of individuals with axSpA or OA diagnoses. Interestingly, similar factors were associated with non-response in both samples: age, sex, being in rheumatologic/orthopedic treatment, having received influenza vaccination, and prescription of physical therapy. Several other studies found similar results regarding age and sex of responders [22,23], but the interaction of these was not addressed. In the Quality and Costs of Primary Care Canadian survey [23] that collected physician and patient reported data in primary care, Li et al., 2018 [23] observed similar results regarding the age and sex of responders: They were older and more often female than non-responders. Opposed to our findings, non-responders had more comorbidities than responders. As the patients for the study by Li et al., 2018 [23] were consecutively sampled in the waiting room, there might be a bias toward persons with higher health care utilization. Another study by Shortreed et al., 2016 [24] used telephone interviews in patients 45 years or older receiving chronic opioid therapy. This study had a response of 59% with the highest response in the age group 65–74 and a higher response in women. Considering comorbidities, persons with a medium number of comorbidities had the highest response. In our samples, responders and non-responders did not differ relevantly in the number of comorbidities as well as in the median number of drugs that can also be used as a proxy for comorbidity. However, in the logistic regression model, comorbidity was associated with a lower response in axSpA.

In our data, there seems to be an overlap of several effects on the response behavior. A higher utilization of influenza vaccination and physical therapy among responders suggest that persons with a higher health awareness were more likely to participate in the survey. In addition, more health care utilization suggesting higher disease burden (medication prescriptions, specialist contact) was found in responders. This is similar to findings of Vercambre and Gilbert (2012) in a survey [25] of unselected persons insured in the national education system health insurance plan. So, there was also a tendency that persons with an elevated disease burden might have had a stronger incentive to actively participate in research. The result that responders more often had physician contact and had more prescriptions of drugs was comparable to results of the Danish national health survey 2010 [22].

Women were more likely to respond in the surveys. This has also been shown in other health-related surveys in Germany [26] and Europe [27] and seems to be a general phenomenon [28]. This might be explained by a higher health awareness that is also reflected by the higher influenza vaccination rates in women [29].

In the Diadec survey on patients with a diagnosis of diabetes in claims data [13], the results were strikingly comparable to our results concerning the age and sex interaction, hospitalization, and medication utilization. In their survey, women were also more likely to answer among the younger persons but not among the older age groups. It is important to note that this effect could be missed if only response by sex was investigated, because the overall response did not differ much. However, considering the interaction, there were relevant differences: young men responded considerably less often than young women. Regardless of the sex, older persons responded more frequently than younger persons. We think it is possible that this interaction is overlooked in studies that do not cover such a broad age spectrum as in the PROCLAIR surveys. A possible reason for younger women participating more often than younger men might be higher health awareness among the women, while elderly men and women seem to have equal health behavior [29].

A large effect of sending a reminder was observed when response times were evaluated. In both samples, the number of responders was doubled after sending the reminder. Sending a reminder seemed to have a similar effect in both surveys, although the time between initially sending the survey and the reminder differed substantially. In the axSpA survey, the reminder was sent after 4 weeks. This seemed to be a little bit too early, because some persons reported feeling pressured to answer, considering the survey had “just been sent”. The response was the same in both surveys, regardless of whether the reminder was sent after 4 or after 12 weeks. Due to the very similar settings of the axSpA and OA surveys, this suggests that there was no influence of the timing of the responder on the response. However, we did not differentiate between initial responders and responders after the reminder. Other data show that there are no relevant differences between participants who reacted to the first mailing of a survey and those who reacted to the reminder [30,31]. Sending a reminder is just one of many possibilities to increase response in a survey. A Cochrane review [32] identified 110 distinct measures to motivate persons to participate in a postal survey. As all of the participants of the PROCLAIR surveys in this manuscript were contacted in the same way, we cannot draw any conclusions about measures that might increase response from this study.

An alternative to postal surveys would have been web-based questionnaires. Kelfve et al. [33] (2020) performed a study where participants were sent a web questionnaire, and a paper questionnaire was offered only with the last reminder. In this study women, retired persons, persons with signs of depression, singles, persons with lower education, and worse health were more likely to fill out the paper questionnaire. Based on these results, we can still recommend the use of paper questionnaires for health-related surveys in order to avoid unnecessary non-response bias.

### 4.1. Implications

Considering the lower response for some age and sex strata, we recommend oversampling younger and male persons for surveys in persons with musculoskeletal diseases. When interpreting results of the PROCLAIR study, readers should always have in mind that there was some non-response bias, especially considering specialist physician contact. Seeing that many effects did not differ much between the axSpA and OA samples suggests that the differences we found in responders and non-responders follow the same mechanisms in different musculoskeletal diseases. The similar results in the Diadec study [13] imply generalizability to other chronic diseases. The effect of a reminder did not depend on the time between sending the first survey and the reminder. Therefore, we recommend finding a balance between very early reminders, which may make persons feel pressured, and very late reminders, which may delay the entire study.

### 4.2. Limitations and Strengths

The diagnoses available from the claims data were not clinically validated diagnoses. For the axSpA sample, we obtained patient confirmations for the diagnosis from those who responded to the survey. In this group, 85% of the responders confirmed their axSpA diagnosis [20], while the remaining 15% mostly reported other rheumatic diagnoses. We had no information about the clinical validity of the claims diagnoses in the non-responders and about socioeconomic status, marital/relationship status, disease severity, or disease activity. However, differences in medication suggest that the survey responders rather had a more severe disease than the non-responders in axSpA, because there were more persons treated with bDMARDS in the responders. However, this difference was not substantial.

Specific strengths of this study were that we could analyze a range of variables in the non-responders, which is not possible in settings where survey participants are recruited differently. Data on comorbidity, medication, age, sex, and health care utilization allowed for a good characterization of survey non-responders, even when information on the severity of disease was missing.

## 5. Conclusions

We have shown that responders and non-responders in two surveys of persons with musculoskeletal diseases differ in certain characteristics. Overall, women responded more often, but not among the age group of 60–79-year-olds in axSpA and the age group of 70–79-year-olds in OA, where men had a higher OR of responding. This fact should be considered when planning sample sizes for comparable surveys. It was also demonstrated that persons in rheumatologic/orthopedist treatment, persons with a prescription of physical therapy, and persons being vaccinated against influenza have a higher OR of responding. It is important to keep in mind that responders might be more strongly affected by the disease than non-responders and that they could have different health behavior. Furthermore, we showed that it can be very effective to send a reminder and that the timing of when to send it does not seem to influence the response much. To sum up, the findings seem robust among musculoskeletal diseases and stress the importance of being aware of non-response bias when planning, conducting, and analyzing a survey.

## Figures and Tables

**Table 1:** Characteristics of responders and non-responders in the axial spondyloarthritis and osteoarthritis sample.

Sample	Axial Spondyloarthritis		Osteoarthritis	
Response status	Responder n = 2082	Non-Responder n = 2389	Responder n = 3564	Non-Responder n = 5420
Age (years), mean	58.3	56.7	67.2	67.5
Women, %	49.9	48.2	70.5	69.0
NSAIDs ^1^, %	57.9	50.1	48.0	39.6
Biologic disease-modifying anti-rheumatic drug, %	13.9	9.0	0.9	0.5
Opioids, %	16.5	14.8	15.2	14.3
Seeing a Rheumatologist, %	32.7	21.1	7.1	3.9
Seeing an Orthopedist, %	39.6	31.3	56.8	43.7
Elixhauser Score (0–31), Median	2.6	2.5	2.8	2.6
Number of prescribed medication, Median	6.0	5.0	6.4	5.8
Influenza vaccination, %	32.5	22.3	39.2	29.0
Hospitalized, %	27.1	25.1	30.9	28.1
Level of care present, %	3.8	4.4	3.1	5.9
Physical therapy, %	50.4	38.7	48.6	40.1
Persons with diagnosis in all quarters of survey year, %	71.9	59.9	58.3	46.5

^1^ NSAID: non-steroidal anti-inflammatory drug.

**Table 2:** Results of one multiple regression model with response as the outcome for each sample. Selected effects of the age and sex interaction are shown.

-Sample	-	-	Axial Spondyloarthritis		Osteoarthritis	
Parameter	Effect	Reference	Odds Ratio (95% CI)	*p*-Value	Odds Ratio (95% CI)	*p*-Value
**Age**	40–49	18–39	1.40 (1.06; 1.85)	0.02	-	-
	50–59		1.73 (1.31; 2.28)	<0.001	-	-
	60–69		1.84 (1.38; 2.45)	<0.001	-	-
	70–79		1.79 (1.33; 2.40)	<0.001	-	-
	40–49	30–39	-	-	1.32 (1.05; 1.66)	0.02
	50–59		-	-	1.67 (1.33; 2.09)	<0.001
	60–69		-	-	1.69 (1.35; 2.12)	<0.001
	70–79		-	-	1.70 (1.34; 2.14)	<0.001
**Sex**	male	female	0.75 (0.56; 1.00)	0.047	0.51 (0.38; 0.67)	<0.001
Age * Sex	18–39, male	18–39, female	Reference	-	-	-
	30–39, male	30–39, female	-	-	Reference	-
	40–49, male	40–49, female	1.06 (0.71; 1.57)	0.78	1.30 (0.90; 1.87)	0.16
	50–59, male	50–59, female	1.03 (0.69; 1.52)	0.89	1.45 (1.02; 2.05)	0.04
	60–69, male	60–69, female	1.47 (0.99; 2.18)	0.06	1.67 (1.19; 2.35)	0.003
	70–79, male	70–79, female	1.83 (1.23; 2.73)	0.003	2.56 (1.83; 3.63)	<0.001
**Elixhauser Comorbidity Index**		per comorbidity	0.95 (0.92; 0.98)	<0.001	1.00 (0.98; 1.04)	0.78
**Opioids**	Yes	No	-	-	1.00 (0.84; 1.21)	0.96
**Biologic disease-modifying anti-rheumatic drug**	Yes	No	1.28 (1.02; 1.59)	0.03	-	-
**In Rheumatologic treatment**	Yes	No	1.71 (1.46; 2.01)	<0.001	-	-
**Orthopedist treating**	Yes	No	-	-	1.56 (1.37; 1.78)	<0.001
**Influenza Vaccination**	Yes	No	1.32 (1.13; 1.55)	<0.001	1.40 (1.22; 1.61)	<0.001
**Level of care present**	Yes	No	0.79 (0.56; 1.11)	0.17	0.43 (0.30; 0.62)	<0.001
**Physical therapy**	Yes	No	1.42 (1.25; 1.62)	<0.001	1.18 (1.03; 1.35)	0.01
**Number of quarters with diagnosis in survey year**		per quarter	1.18 (1.12; 1.25)	<0.001	1.04 (1.00; 1.09)	0.07

CI: confidence interval. Empty cells: Parameter is not included in the model by predefined choice.

## Supplementary Materials

The following are available online at https://www.mdpi.com/1660-4601/17/24/9186/s1, Table S1: Comparison of women and men in the age groups.
