# Supplementary material for: Factors Associated with Survey Non-Response in a Cross-Sectional Survey of Persons with an Axial Spondyloarthritis or Osteoarthritis Claims Diagnosis
Source: Int J Environ Res Public Health. 2020 Dec 9;17(24):9186. doi: 10.3390/ijerph17249186 (PMC7764396; doi:10.3390/ijerph17249186)
Supplement: Supplementary file 1 [file ijerph-17-09186-s001.pdf]

Supplementary table S1

	Axial Spondyloarthritis		Osteoarthritis	
**Contrast**	Odds Ratio (95% Confidence Interval)	p-value	Odds Ratio (95% Confidence Interval)	p-value
**18-39, female vs male**	1.34 (1.00; 1.79)	0.047	-	-
**30-39, female vs male**	-	-	1.97 (1.48; 2.60)	<0.001
**40-49, female vs male**	1.27 (0.96; 1.67)	0.09	1.51 (1.20; 1.91)	<0.001
**50-59, female vs male**	1.30 (1.00; 1.70)	0.05	1.36 (1.11; 1.67)	0.004
**60-69, female vs male**	0.91 (0.70; 1.20)	0.51	1.18 (0.97; 1.43)	0.10
**70-79, female vs male**	0.73 (0.56; 0.97)	0.03	0.76 (0.63; 0.93)	0.006

Odds ratios from the model in table 2 that show comparisons of the odds ratio to respond in female vs. male subjects given a certain age group.
